# Exploring the Role of Self-Efficacy in Maintaining Healthy Lifestyle Habits among Patients with Cardiometabolic Diseases; Findings from the Multi-Center IACT Cross-Sectional Study

**DOI:** 10.3390/life14060736

**Published:** 2024-06-07

**Authors:** Vasiliki Kalantzi, Thomas Tsiampalis, Matina Kouvari, Vasiliki Belitsi, Antonios Zairis, Athanasios Migdanis, Sousana K. Papadopoulou, Fotini Bonoti, Demosthenes B. Panagiotakos, Rena I. Kosti

**Affiliations:** 1 Department of Nutrition and Dietetics, School of Physical Education, Sports and Dietetics, University of Thessaly, 38221 Trikala, Greece; vkalantzi@uth.gr (V.K.); ttsiampalis@uth.gr (T.T.); mkouvari@uth.gr (M.K.); vbelitsi@uth.gr (V.B.); amigdanis@uth.gr (A.M.); fbonoti@uth.gr (F.B.); 2 Department of Nutrition and Dietetics, School of Health Science and Education, Harokopio University, 17676 Athens, Greece; dbpanag@hua.gr; 3 Faculty of Health, University of Canberra, Canberra, ACT 2617, Australia; 4 Department of Economics and Business, School of Economics, Business and Computer Sciences, Neapolis University, Paphos 8042, Cyprus; a.zairis@nup.ac.cy; 5 Department of Nutritional Sciences and Dietetics, School of Health Sciences, International Hellenic University, 57400 Thessaloniki, Greece; sousana@the.ihu.gr

**Keywords:** cardiometabolic patients, self-efficacy, adherence, lifestyle behaviors, Mediterranean diet, smoking, physical activity

## Abstract

(1) Background: Cardiometabolic disease progression can be delayed if patients engage in healthy lifestyle behaviors, adherence to which is highly influenced by psychosocial factors. The present study aimed at investigating the association of self-efficacy with the adherence level to healthy lifestyle behaviors among patients with cardiometabolic diseases in Greece. (2) Methods: 1988 patients (1180 females) with cardiometabolic diseases participated. Anthropometric, demographic, socioeconomic, clinical, and lifestyle characteristics were recorded. Patients were also asked to evaluate their efficacy to comply with healthy lifestyle behaviors. (3) Results: The majority exhibited unhealthy lifestyle behaviors. A subgroup demonstrated elevated self-efficacy in maintaining healthy habits despite facing diverse psychosocial challenges. Individuals with higher educational attainment, socioeconomic status, and rural/semi-urban residency had significantly elevated self-efficacy. Those with heightened self-efficacy exhibited significantly lower BMI and reduced prevalence of certain health conditions. Self-efficacy significantly influenced adherence to the Mediterranean diet, physical activity engagement, and smoking cessation, even in challenging circumstances. (4) Conclusions: This study represented an innovative approach in examining the role of self-efficacy in shaping health behaviors and outcomes within a Greek population. By integrating specific psychosocial circumstances into the analysis, valuable insights were provided into the contextual factors influencing self-efficacy and adherence to healthy lifestyle behaviors.

## 1. Introduction

Cardiometabolic diseases (CMDs), encompassing conditions such as cardiovascular disease, type 2 diabetes, and metabolic syndrome, constitute a significant public health concern globally, including in Greece [1,2]. Recognizing the multifaceted nature of CMDs, efforts have been directed towards not only treating established cases but also towards preventive measures aimed at reducing risk factors and promoting healthy lifestyle behaviors. These endeavors encompass a wide range of strategies, including public health campaigns, policy interventions, and clinical initiatives, all aimed at mitigating the burden of CMDs on individuals and healthcare systems. Lifestyle factors, including dietary habits, physical activity levels, and smoking, play a pivotal role in the development and management of cardiometabolic conditions [3]. As it can be seen in the latest clinical practice guidance by the European Society of Cardiology, the Mediterranean diet stands out for its well-documented health benefits and alignment with dietary guidelines, making it a suitable focus for our investigation [4]. Adherence to a healthier lifestyle, such as adopting the Mediterranean diet, engaging in regular physical activity, and abstaining from smoking, has been associated with improved cardiometabolic outcomes and reduced disease risk [5,6]. However, despite the well-documented benefits of these behaviors, achieving and maintaining adherence can be challenging for many individuals, particularly those grappling with psychosocial stressors and environmental barriers [7,8,9].

In this context, the concept of self-efficacy, i.e., the individuals’ belief in their ability to successfully execute specific behaviors to achieve desired outcomes, encompassing confidence in overcoming obstacles and persisting in the face of setbacks [10], emerges as a central determinant of health behavior change and maintenance [11]. Previous studies revealed that individuals are unlikely to engage in a specific behavior if they lack confidence in their ability to perform it [12]. Accordingly, it has been demonstrated that higher levels of self-efficacy are associated with greater adherence to dietary recommendations, increased engagement in physical activity, and lower smoking rates [13,14,15]. Despite these promising findings, there remains a need for further research to examine the specific role of self-efficacy in shaping adherence to healthier lifestyle practices among patients with cardiometabolic diseases in Greece, where cultural and environmental factors may influence behavior patterns differently. In particular, cultural norms, such as communal dining and traditional recipes being passed down through generations, as well as environmental conditions—such as access to healthy local markets and neighborhood shops; pedestrian-friendly streets; and outdoor recreational spaces encouraging walking, cycling, and other forms of exercise—can significantly impact how individuals behave, including the adoption of healthy lifestyle practices [16,17]. Thereby, the aim of the present study was to investigate the association of self-efficacy in terms of complying with healthy lifestyle behaviors, even in challenging psychosocial circumstances, with the level of adherence to the Mediterranean diet, engagement in frequent physical activity, and absenteeism from smoking, among patients with cardiometabolic diseases in Greece. In particular three a priori research hypotheses were performed: patients with higher levels of self-efficacy demonstrate a. greater adherence to the Mediterranean diet, even when encountering challenges such as household members consuming non-dietary foods or experiencing stress and negative emotions, b. increased engagement in physical activity, notwithstanding constraints such as time limitations due to multiple obligations or stressors, and c. lower smoking rates, even in situations where they are exposed to triggers such as social events away from home. While it is true that the issue we are addressing has persisted despite available solutions, our study aims to delve deeper into the underlying factors contributing to this ongoing challenge. By investigating the association between self-efficacy and adherence to healthy lifestyle behaviors among patients with cardiometabolic diseases in Greece, we seek to provide novel insights that could potentially enhance existing strategies and interventions for addressing this longstanding issue.

## 2. Materials and Methods

### 2.1. Study Design and Scope

The Integrated Assessment of Adherence to Treatment Questionnaire for CardioMetabolic Diseases (IACT) is a cross-sectional study conducted in Greece during 2022–2023.

### 2.2. Study Setting

The research took place in 7 regions of Greece (Attica, Thessaly, Aegean, Crete, Epirus and West Macedonia, Peloponnese and West Greece, East Macedonia, and Thrace) where participants had visited and received treatment from primary care settings. Recruitment also took place in the medical facilities of 1st Multipurpose Municipal Clinic of Solonos Athens, 2nd Multipurpose Municipal Clinic of Neos Kosmos, 3rd Municipal Clinic of Petralona, 4th Municipal Clinic of Kolonou, 6th Multipurpose Municipal Clinic of Kypseli, Trikala—Farkadona Medical Center, Pyli Medical Center, and Kalambaka Medical Center, according to the researchers’ accessibility. Areas in the region of Thessaly were considered as rural/semi-urban areas, as the majority of its population is considered agrarian and semi-urban [18].

### 2.3. Sample Size

This cross-sectional study involved the enrollment of 1988 participants who had been diagnosed with cardiovascular diseases (CVDs) (specifically, coronary heart disease (CHD) and stroke) or other cardiometabolic conditions, such as hypertension, type 2 diabetes, type 1 diabetes, hypercholesterolemia, elevated triglycerides, obesity, and non-alcoholic fatty liver disease, out of whom 1180 were females. These individuals were recruited from various healthcare facilities in the specified regions using a convenience sampling approach at different days and times in order to reduce the bias, where participants meeting the eligibility criteria were consecutively enrolled until the desired sample size was achieved. Participants were chosen from several different urban and semi-urban areas of Greece in order to represent the different socioeconomic and demographic profiles of this population. This approach ensures a structured and consistent method of participant selection, reducing potential biases associated with the convenience sampling method. The average response rate across the different healthcare facilities exceeded 70%, with the primary reasons for non-participation being the necessity of attending medical appointments and the lack of affiliation between the research team and the medical centers.

### 2.4. Eligibility Criteria

All participants met the following eligibility criteria: (1) were of Greek nationality; (2) were at least 18 years of age; (3) had received treatment from primary healthcare settings; (4) had received medical diagnosis for at least one of the following diseases: hypertension, type II diabetes, type I diabetes, hypercholesterolemia, elevated triglycerides, obesity, coronary heart disease, stroke, non-alcoholic fatty liver disease; (5) were prescribed and taking one or more medications for cardiometabolic diseases for more than a year.

### 2.5. Bioethics

The present study adheres to the ethical guidelines of the Declaration of Helsinki and has been approved by the Ethics Committee of the University of Thessaly, Department of Nutrition and Dietetics (Ethics 11-14/07/2022). All participants were informed about the objectives and procedures of the study, and they provided their written consent to participate.

### 2.6. Research Tool

The Integrated Assessment of Adherence to Treatment Questionnaire for CardioMetabolic Diseases (IAATQ-CMD) research tool, which was found to be reliable and repeatable [19], was used, as outlined in the study of Belitsi et al. [19], by experienced healthcare professionals in order to ensure that all participants fully comprehended and accurately responded to each of the questions. The study questionnaire was pilot tested with 10 healthcare professionals in primary care settings, and improvement modifications were made. Further details concerning the procedures can be found elsewhere [19].

#### 2.6.1. Anthropometric and Socio-Demographic Data

The study evaluated participants’ socio-demographic features, encompassing education level, occupation, income, marital status, age, gender, nationality, and area of residence. To elaborate, participants’ education level varied from primary school to PhD and beyond, categorized into Group I (primary education), Group II (secondary education), and Group III (higher tertiary education). Occupational status divided participants into employed/freelance (Group I) and unemployed/retired (Group II). Income was classified as low (<18,000 euros/year) or moderate (≥18,000 euros/year), aligning with OECD’s average household net-adjusted disposable income per capita threshold in Greece. Marital status, age, gender, and nationality were also recorded. Height and weight were self-reported, enabling the calculation of body mass index (BMI), with individuals exceeding 29.9 kg/m^2^ categorized as obese. A detailed questionnaire captured comprehensive medical history, encompassing cardiovascular risk factors and pre-existing health conditions. Further details are available in the study of Belitsi et al. [19].

#### 2.6.2. Medical History

The patients’ medical history was obtained through a series of questions that entailed cardiometabolic diseases and risk factors that had been diagnosed and treated by a doctor for more than a year, along with other pre-existing health conditions [19].

#### 2.6.3. Dietary and Lifestyle Data

Adherence to the Mediterranean diet was measured using the MedDietScore, a scale ranging from 0 to 55 [20]. This assessment focused on 11 essential components of the Mediterranean diet, such as non-refined cereals, fruits, vegetables, potatoes, legumes, olive oil, fish, red meat, poultry, full-fat dairy products, and alcohol. Scores between 0 and 5 were assigned based on the frequency of consumption, with 0 indicating no consumption and 5 indicating daily consumption. Conversely, scores for items not conforming to the diet were assigned in reverse order within the same range. Alcohol consumption was scored differently, with 5 assigned for consuming less than 300 mL per day, 0 for exceeding 700 mL per day or no consumption, and scores between 4 and 1 for consumption levels between 300–400 mL, 400–500 mL, 500–600 mL, and 600–700 mL per day (where 100 mL equates to 12 g of ethanol). The overall score, ranging from 0 to 55, was then calculated. Visual references from the “National Dietary Guidelines for Adults” in Greece were incorporated to illustrate food portions, aiming to improve clarity, comprehension, and consistency in depicting serving sizes [21]. Higher total scores indicated stronger adherence to the Mediterranean diet, and patients were categorized as low or high adherers based on the median value of 31 units on the scale.

Regarding lifestyle factors, participants were questioned about their physical activity levels and smoking habits. They were asked to indicate their exercise status (yes/no), specify the frequency (1–2 times per week, 3–4 times per week, at least 5 times per week), and type of exercise (e.g., low-intensity walking; cycling; Pilates; yoga; high-intensity activities like jogging, swimming, dancing, tennis, gardening). Additionally, participants disclosed their smoking status, including current, past, or passive smoking, with responses provided in a yes/no format. A scoring index was devised to gauge patients’ adherence to the Mediterranean diet (high/low), their level of physical activity, and their smoking habits, collectively termed as their “healthy lifestyle”. Patients were classified as having at least one unhealthy parameter and having no unhealthy parameters.

#### 2.6.4. Assessment of Self-Efficacy in Terms of Compliance to Healthy Lifestyle Behaviors

Self-efficacy in terms of compliance to healthy lifestyle behaviors was estimated via 6 questions designed to assess patients’ confidence of their ability to comply with healthy lifestyle behaviors under challenging psychosocial circumstances. More specifically, questions in the form of a Likert scale required the participants to rate their confidence in complying to healthy lifestyle habits when: (i) other people in the house consume food that is not part of their diet, (ii) others drink with no control, (iii) they are stressed or have negative feelings, (iv) they are outside of the house, (v) their partner/friends smoke, as well as (vi) they are burdened with many obligations and do not have time to exercise. All responses were in the form of “not at all/slightly/moderately/enough/very”. Based on their answers, patients were categorized as being very and at most enough confident about adhering to healthy lifestyle habits under each circumstance. In addition, a composite score was also created, in which higher values indicated higher self-efficacy levels in terms of compliance to healthy lifestyle behaviors (range: 0–24). Patients were also categorized as having low or high self-efficacy in terms of complying to healthy lifestyle behaviors based on the median value of 19 units on this scale.

### 2.7. Statistical Analysis

The patients’ categorical characteristics are depicted as percentages (%), while continuous variables are represented by mean values with their respective standard deviations (SD). The normality assumption of continuous variables’ distributions was assessed using graphical methods (such as histograms, PP-plots, and QQ-plots) as well as the Shapiro–Wilk test. The independent samples *t*-test was employed to explore the relationship between patients’ continuous traits, their lifestyle type (healthy/unhealthy), and their self-efficacy level (low/high), while Pearson’s chi-square test was utilized to investigate associations with categorical patient characteristics. In addition, the one-way analysis of variance (ANOVA) was employed in order to examine the difference in self-efficacy levels (Continuous form) among the three educational levels, stratified by the patients’ area of residence (rural/semi-urban and urban) and sex (males/females).

To examine the link between patients’ lifestyle attributes (adherence to the Mediterranean diet, smoking status, physical activity level) and their self-efficacy across various situations, a multivariable binomial logistic regression analysis was conducted. Results are presented as odds ratios (OR) with corresponding 95% confidence intervals (CI). These logistic regression models were adjusted for patients’ demographic factors (age, sex), socioeconomic factors (educational level), and clinical characteristics (number of chronic conditions). All statistical analyses were performed using SPSS v29.0, with statistical significance set at a *p*-value of <0.05 for two-tailed tests.

## 3. Results

### 3.1. Participants’ Characteristics Based on Their Lifestyle Status

Table 1 displays the participants’ characteristics in the IACT study, categorized by the number of unhealthy parameters (low adherence to the Mediterranean diet or sedentary lifestyle or smoking). As depicted, the vast majority of the patients (75.9%) exhibited unhealthy lifestyle behaviors, such as diverging from the Mediterranean diet, smoking, or maintaining a sedentary lifestyle. The data indicate that individuals with a healthy lifestyle tended to be younger (*p* = 0.001), possessed higher socioeconomic levels in terms of higher educational levels (*p* < 0.001), better occupational status (*p* < 0.001), and higher income levels (*p* = 0.001), while they also had a higher probability of living in rural/semi-urban areas (*p* < 0.001). In terms of clinical attributes, participants with a healthy lifestyle demonstrated significantly lower BMI (*p* < 0.001). Additionally, within this group, there was a lower prevalence of hypertension (*p* = 0.033), type 1 diabetes mellitus (*p* = 0.024), hypercholesterolemia (*p* < 0.001), and kidney disease (*p* = 0.001).

### 3.2. Participants’ Characteristics Based on Their Level of Self-Efficacy under Challenging Psychosocial Circumstances

Based on the composite score created, it emerged that approximately half of the patients (52.9%) were classified as having elevated self-efficacy levels in terms of complying with healthy lifestyle behaviors despite encountering diverse challenging psychosocial circumstances. As outlined in Table 2, those with high self-efficacy tended to be older (*p* < 0.001) and possessed higher educational attainment (*p* < 0.001), along with enjoying a higher socioeconomic status in terms of both occupational status and income level and were more likely to reside in rural/semi-urban areas. Regarding clinical attributes, participants exhibiting high self-efficacy demonstrated notably lower BMI (*p* < 0.001). Moreover, within this cohort, there was a reduced prevalence of hypertension (*p* < 0.001), type 2 diabetes mellitus (*p* < 0.001), hypercholesterolemia (*p* < 0.001), elevated triglyceride levels (*p* < 0.001), and NAFLD (*p* < 0.001). Conversely, among patients with high self-efficacy in complying with healthy lifestyle behaviors despite challenging psychosocial circumstances, the prevalence of kidney disease was significantly higher (*p* = 0.018).

**Figure 1:**
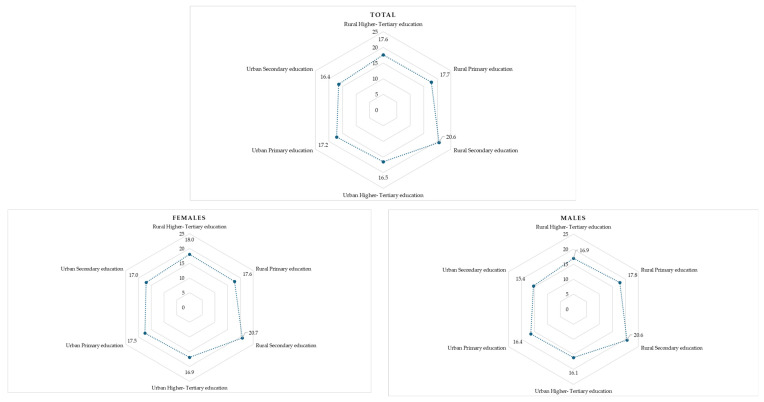
Patients’ self-efficacy levels in terms of complying with healthy lifestyle behaviors, stratified by their sex, area of residence, and educational level.

Figure 1 presents the self-efficacy levels of patients in terms of their compliance with healthy lifestyle behaviors, classified by gender, residential area, and educational level. Overall, individuals living in rural/semi-urban areas tended to have higher self-efficacy levels compared to those in urban areas, regardless of gender [Mean (SD): Rural/Semi- urban 19.5 (5.7), Urban 16.6 (5.4); *p* < 0.001]. Among rural/semi-urban residents, the highest self-efficacy levels were observed among patients with a secondary educational level [Mean (SD): Primary education 17.7 (6.8), Secondary education 20.6 (4.8), Higher-Tertiary education 17.6 (6.3)], while among urban dwellers, no significant difference was observed across the three educational levels (*p* = 0.102). This pattern varied notably between genders (interaction *p*-value < 0.001). Specifically, among females in rural/semi-urban areas, those with lower educational levels displayed the lowest self-efficacy levels [Mean (SD): Primary education 17.6 (6.7), Secondary education 20.7 (4.9), Higher-Tertiary education 18.0 (5.9); *p* < 0.001], whereas among females in urban areas, no significant discrepancy was noted among the three educational levels.

### 3.3. Self-Efficacy and Level of Adherence to the Mediterranean Diet

As outlined in Table 3, patients with high self-efficacy in terms of complying to healthy lifestyle behaviors under various psychosocial circumstances had 72% higher odds of being classified as high adherers to the Mediterranean diet, compared to those with low self-efficacy (OR = 1.72, 95%CI = 1.45–2.04). More specifically, those maintaining high confidence for complying to healthy lifestyle behaviors, even in social situations, exhibited 1.6 times greater odds of being classified as high adherers to the Mediterranean diet (OR = 1.61, 95%CI = 1.33–1.92), while patients demonstrating confidence for complying to healthy lifestyle behaviors despite time constraints had approximately 1.5 times greater odds (OR = 1.45, 95%CI = 1.20–1.72). Moreover, individuals with high self-efficacy, even when others excessively consume alcohol, showed a 35% increase in the odds of being classified as high adherers to the Mediterranean diet (OR = 1.35; 95%CI = 1.12–1.61). Similarly, those being characterized by high confidence in terms of complying to healthy lifestyle behaviors, even when others eat foods that are not part of their diet (OR = 1.25, 95%CI = 1.04–1.49) or when they have stress and negative feelings (OR = 1.27, 95%CI = 1.05–1.52), had at least 25% higher odds of being classified as high adherers to the Mediterranean diet.

### 3.4. Self-Efficacy and Physical Activity Status

As depicted in Table 4, patients with high self-efficacy in terms of complying to healthy lifestyle behaviors under various psychosocial circumstances had 30% higher odds of being physically active, compared to those with low self-efficacy (OR = 1.30, 95%CI = 1.09–1.56). In particular, patients who reported confidence for complying with healthy lifestyle behaviors, even when experiencing stress and negative emotions, exhibited notably higher levels of physical activity. Specifically, individuals demonstrating confidence despite stress and negative feelings had 1.25 times higher odds of being physically active (OR = 1.25, 95%CI = 1.04–1.50), while at the same time, patients who maintained confidence in terms of complying to healthy lifestyle behaviors, even when their life was burdened with numerous obligations and they lacked time for exercise, had approximately 20% higher odds of being physically active (OR = 1.19, 95%CI = 1.01–1.43).

### 3.5. Self-Efficacy and Smoking

Table 5 illustrates a significant association between elevated levels of patients’ self-efficacy regarding compliance with healthy lifestyle behaviors and a reduced propensity for smoking. Specifically, individuals with strong self-efficacy in terms of complying with healthy lifestyle behaviors across various psychosocial situations were nearly twice as likely to be nonsmokers compared to those with lower self-efficacy (OR = 1.98, 95% CI = 1.64–2.40). Patients exhibiting high confidence in maintaining healthy lifestyle habits, even in the presence of a smoking partner or friend, showed nearly six times greater odds of being nonsmokers compared to those with lower confidence (OR = 5.88, 95% CI = 5.00–7.69). In other psychosocial scenarios, individuals with high self-efficacy in complying with healthy lifestyle behaviors had at least a 32% higher chance of being nonsmokers, as demonstrated in the data presented.

## 4. Discussion

The current investigation explored the intricate nexus between lifestyle behaviors, self-efficacy, and health outcomes within the cohort of participants in the IACT study. It was evident that a notable percentage of individuals exhibited unhealthy lifestyle practices, characterized by suboptimal adherence to the Mediterranean diet, smoking habits, and sedentary tendencies. However, amidst this concerning trend, a distinct subgroup emerged, individuals displaying heightened levels of self-efficacy in maintaining healthy lifestyle behaviors despite encountering diverse psychosocial challenges. These individuals showcased resilience, demonstrating a steadfast belief in their capacity to enact positive changes in their lives. Notably, they demonstrated higher educational attainment and socioeconomic status, indicating the influence of access to resources and knowledge on shaping self-efficacy. Interestingly, rural/semi-urban residency was associated with higher levels of self-efficacy, suggesting variations in social and environmental contexts between urban and rural/semi-urban settings that may impact health behavior beliefs. Additionally, individuals with heightened self-efficacy exhibited lower BMI and reduced prevalence of certain health conditions, underscoring the potential impact of self-belief on health outcomes. Remarkably, this group shared similarities with individuals leading healthier lifestyles, hinting at a potential synergy between self-efficacy and positive health outcomes. Our analysis further accentuated the profound influence of self-efficacy on specific health behaviors, such as adherence to the Mediterranean diet, engagement in physical activity, and smoking cessation. Furthermore, our study meticulously explored the influence of specific psychosocial circumstances on adherence to healthy lifestyle behaviors. Individuals with elevated self-efficacy levels demonstrated significantly higher odds of adhering to the Mediterranean diet, even in challenging scenarios such as social gatherings where others consumed alcohol or non-dietary foods, or during periods of stress and negative emotions. Moreover, those with heightened self-efficacy displayed greater involvement in physical activity, even in the face of time constraints or stressful situations.

The association between higher levels of educational attainment, socioeconomic status, and elevated self-efficacy observed in our study suggests the pivotal role of access to resources and knowledge in shaping individuals’ beliefs and attitudes towards health behaviors. Individuals with greater educational and socioeconomic advantages may have increased access to health information, resources, and supportive networks, thereby fostering a stronger sense of self-efficacy in adopting and maintaining healthy lifestyle behaviors [22]. Conversely, the finding that rural/semi-urban residency was associated with higher levels of self-efficacy implies that social and environmental contexts play a significant role in shaping health behavior beliefs [23]. Rural areas may offer distinct social support systems, community norms, and access to natural environments conducive to physical activity, which could contribute to higher levels of self-efficacy among residents [24]. Additionally, rural communities may exhibit tighter-knit social networks and shared cultural values that promote collective efficacy, further bolstering individuals’ confidence in their ability to engage in health-promoting behaviors [25].

Our findings agree with prior studies outlining the significance of self-efficacy and confidence in patients’ ability to adhere to the recommended health regime. More specifically, self-efficacy may influence dietary choices by enhancing individuals’ belief in their ability to overcome challenges such as social pressure or time constraints. Research centered on the CVD population has highlighted the constructive impact that self-efficacy has on individuals when it comes to embracing a healthy dietary regime [26,27] or to committing to dietary changes [13,28]. Self-efficacy may empower individuals to resist unhealthy food temptations and adhere to dietary recommendations, leading to improved dietary habits and health outcomes. A wide range of studies have concluded that the more self-efficient patients are, the more effective they are in adapting to the recommended lifestyle changes and tackling their disease [29,30,31,32].

When it comes to the Mediterranean diet, self-efficacy is critical in promoting and maintaining adherence, even in challenging circumstances. Patients who maintain their confidence in various situations, such as social events or time constraints, demonstrate higher odds of adhering to the Mediterranean diet [30]. Inversely, it has been proven that individuals that adhere to the Mediterranean diet demonstrate higher motivation, positive mood along with low depression patterns, and overall well-being [33]. In the study of Walker et al., it was also argued that self-efficiency is positively correlated with intrinsic motivation, thus outlining its profound role in prevailing over obstacles [34]. Similarly, Bas et al. outlined the profound role of self-efficacy in obesity treatment when it comes to weight control behavior, as obese participants with low self-efficacy demonstrated lower weight control management due to their inability to control their eating behaviors [35].

In the context of physical activity engagement, studies suggest that self-efficacy impacts individuals’ motivation, goal setting, and persistence in overcoming barriers to exercise [36,37]. Despite facing challenges like stress and time limitations, individuals with higher self-efficacy levels are more likely to engage in regular physical activity [38,39]. This highlights the role of self-efficacy in promoting an active lifestyle, which is essential for overall health and well-being. In a survey about physical activity among middle-aged adults with cardiovascular disease, it was found that self-efficacy was a substantial predictor in determining who will engage in exercise [40]. What is more, higher levels of self-efficacy may enable individuals to maintain a positive outlook, set realistic exercise goals, and persevere in the face of challenges, ultimately leading to increased physical activity levels and improved fitness. This is also evident in the study of McAuley et al., where high self-efficacy was highly correlated with lower levels of fatigue and psychological distress as well as with higher positive mood and greater well-being [41].

Regarding smoking cessation, research indicates that self-efficacy influences individuals’ confidence in their ability to resist smoking triggers and overcome nicotine cravings [42]. In a survey regarding smoking cessation among cardiac patients, it was found that self-efficacy is a good predictor of the intention to quit smoking and indirectly mediates abstinence [43]. The findings suggest that self-efficacy plays a critical role in reducing smoking rates, particularly in challenging circumstances such as social events or exposure to smoking peers [44,45].

Our study is in alignment with previous research, blatantly indicating that higher self-efficacy levels enhance individuals’ coping skills, self-control, and determination to adopt healthy lifestyle changes that include adherence to the Mediterranean diet, adjusting alcohol consumption, cessation of smoking, and engagement in regular physical activity. It is also demonstrated that self-efficacy plays a crucial role in overcoming barriers and maintaining healthy lifestyle habits that ultimately contribute to better health outcomes. According to Bandura, self-efficacy is defined as the perception that individuals have about their own ability to effectuate a goal [12]. In this concept, the social cognitive theory suggests that positive behavioral engagement can be accomplished solely by the positive perception of self-efficacy [12]. In our case, self-efficacy plays a pivotal role in patients with cardiometabolic diseases in terms of adoption and adherence to a healthy lifestyle protocol, as patients who display low confidence in their ability to commit to this health behavioral change are not likely to be able to carry out this change. Thus, patients that are confident in their ability to adhere to their prescribed health regime are more likely to stay consistent and ultimately ameliorate their health.

Overall, this research underscores the considerable impact of self-efficacy on fostering healthier lifestyle habits among individuals with cardiometabolic diseases in Greece, a Mediterranean country where diverse cultural and environmental factors may influence behavior patterns in distinct ways. It is through building confidence and defense mechanisms towards temptations and negative emotions that self-efficacy can regulate eating patterns and play a substantial role in these patients adopting healthy lifestyle behaviors. By understanding the role of self-efficacy in dietary adherence, physical activity engagement, and smoking absenteeism, healthcare professionals can develop targeted interventions to empower patients and improve their long-term health outcomes.

The findings of our study hold important clinical implications for the management and prevention of cardiovascular diseases and other cardiometabolic conditions. Firstly, the identification of individuals with heightened self-efficacy in maintaining healthy lifestyle behaviors despite psychosocial challenges underscores the importance of assessing and fostering self-belief in clinical practice. Interventions aimed at enhancing self-efficacy through targeted counseling, behavioral therapy, and empowerment programs may offer promising avenues for promoting healthier lifestyles and reducing the risk of cardiometabolic diseases among at-risk populations. Additionally, our results highlight the need for tailored interventions that consider the socio-economic and environmental factors influencing self-efficacy and health behaviors. Healthcare providers should strive to create supportive environments that facilitate the adoption and maintenance of healthy lifestyle practices, particularly among individuals with limited access to resources or living in urban areas with unfavorable social determinants of health. By addressing these factors and empowering individuals to overcome barriers to behavior change, clinicians can play a pivotal role in improving health outcomes and reducing the burden of cardiovascular diseases in communities.

## 5. Conclusions

In conclusion, our study sheds light on the role of self-efficacy in promoting healthier lifestyle behaviors among individuals with cardiometabolic diseases in Greece, while also highlighting the investigation of specific psychosocial circumstances as an innovative aspect of our research. Other behaviors—beyond the ones investigated herein—such as sleep quality, should also also considered on the basis of a similar research hypothesis in the near future. Despite a notable prevalence of unhealthy lifestyle practices, individuals displaying heightened levels of self-efficacy showcased resilience and a steadfast belief in their capacity to enact positive changes in their lives. Notably, these individuals tended to have higher educational attainment and socioeconomic status, suggesting the pivotal role of access to resources and knowledge in shaping self-efficacy beliefs. Additionally, rural/semi-urban residency was associated with higher levels of self-efficacy, indicating variations in social and environmental contexts that may impact health behavior beliefs. Our findings align with prior research highlighting the significance of self-efficacy in dietary adherence, physical activity engagement, and smoking cessation among patients with cardiometabolic diseases. Through tailored interventions aimed at bolstering self-efficacy, healthcare professionals can empower patients to overcome barriers and maintain healthier lifestyle habits, ultimately improving long-term health outcomes. By recognizing the central role of self-efficacy in behavior change, along with the innovative exploration of specific psychosocial circumstances, we can pave the way towards a healthier future for individuals with cardiometabolic diseases in Greece and beyond.

## 6. Limitations

Results were obtained from specific regions of Greece, according to the investigators’ accessibility, thus possibly limiting the applicability of the results to the vast Greek population. Also, the questionnaire was administered while the patients were in primary care settings while waiting for treatment for their cardiometabolic disease(s), which may have resulted in patients’ overestimating their self-efficacy and their intention to adhere to the therapeutic lifestyle recommendations. Another limitation is the reliance on self-reported data, which can lead to over or underestimation of patients’ behavioral patterns. Furthermore, while our results suggest a potential relationship between higher self-efficacy levels and healthier lifestyle behaviors, including adherence to the Mediterranean diet, smoking cessation, and engagement in regular physical activity, it is important to interpret these findings with caution. Further research is warranted to elucidate the causal pathways and underlying mechanisms driving these associations. Additionally, future studies should employ more rigorous study designs, such as longitudinal or intervention studies, to establish causality and explore potential mediators and moderators of the observed relationships. By adopting a more cautious and evidence-based approach in our discussion, we aim to contribute to the scientific discourse surrounding self-efficacy and health behavior change among patients with cardiometabolic diseases. Despite these limitations, this study develops a better understanding of the impact of self-efficacy on health behavior changes and could provide the foundation for future randomized controlled trials.

## Figures and Tables

**Table 1:** Patients’ demographic, socioeconomic, anthropometric, and clinical characteristics according to their type of lifestyle; the multi-center IACT study (2022–2023).

	Number of Unhealthy Lifestyle Parameters		*p*-Value
	None	At Least One	
	*N* = 479	*N* = 1509	
Demographic characteristics			
Sex (% Female)	57.6	59.9	0.375
Age [years; Mean (SD)]	63.3 (13.1)	65.7 (14.2)	0.001
Educational level (% Primary education)	16.6	21.1	<0.001
Socioeconomic characteristics			
Occupational status (% Employed)	67.1	44.5	<0.001
Marital status (% Married/Cohabitation)	67.0	66.7	0.909
Income level (% More than 18,000 euros/year)	35.3	27.6	0.001
Area of residence (% Rural/Semi-urban)	72.9	53.5	<0.001
Anthropometric and clinical characteristics			
Body mass index [kg/m^2^; Mean (SD)]	27.0 (3.8)	28.1 (4.1)	<0.001
Hypertension (% Yes)	56.1	61.6	0.033
Type 2 diabetes mellitus (% Yes)	18.0	20.7	0.184
Type 1 diabetes mellitus (% Yes)	2.7	4.8	0.024
Hypercholesterolaemia (% Yes)	33.1	49.1	<0.001
Elevated triglyceride levels (% Yes)	17.3	19.4	0.291
Kidney disease (% Yes)	2.9	7.0	0.001
Non-alcoholic fatty liver disease (% Yes)	5.6	6.3	0.607
Coronary heart disease (% Yes)	17.7	17.7	0.979

Notes: *p*-value was based on Pearson’s chi-square test (in the case of categorical characteristics) and on the independent samples *t*-test (in the case of continuous characteristics); the following unhealthy lifestyle parameters were considered: low adherence to Mediterranean diet, sedentary lifestyle, smoking; Abbreviations—SD: Standard Deviation.

**Table 2:** Patients’ demographic, socioeconomic, anthropometric, and clinical characteristics according to their level of self-efficacy in terms of complying to healthy lifestyle behaviors; the multi-center IACT study (2022–2023).

	Level of Self-Efficacy in Terms of Complying to Healthy Lifestyle Behaviors		*p*-Value
	Low	High	
	*N* = 936	*N* = 1052	
Demographic characteristics			
Sex (% Female)	57.4	61.1	0.089
Age [years; Mean (SD)]	61.9 (14.3)	65.6 (12.3)	<0.001
Educational level (% Primary education)	19.1	16.3	<0.001
Socioeconomic characteristics			
Occupational status (% Employed)	56.6	66.1	<0.001
Marital status (% Married/Cohabitation)	62.6	70.5	<0.001
Income level (% More than 18,000 euros/year)	23.0	36.8	<0.001
Area of residence (% Rural/Semi-urban)	27.1	54.9	<0.001
Anthropometric and clinical characteristics			
Body mass index [kg/m^2^; Mean (SD)]	28.2 (4.4)	27.5 (3.6)	<0.001
Hypertension (% Yes)	65.3	50.4	<0.001
Type 2 diabetes mellitus (% Yes)	24.8	13.1	<0.001
Type 1 diabetes mellitus (% Yes)	3.1	3.3	0.773
Hypercholesterolaemia (% Yes)	49.0	26.2	<0.001
Elevated triglyceride levels (% Yes)	25.3	11.1	<0.001
Kidney disease (% Yes)	4.7	7.2	0.018
Non-alcoholic fatty liver disease (NAFLD; % Yes)	9.2	2.8	<0.001
Coronary heart disease (% Yes)	17.8	17.6	0.881

Notes: *p*-value was based on Pearson’s chi-square test (in the case of categorical characteristics) and on the independent samples *t*-test (in the case of continuous characteristics); patients were categorized as having low or high self-efficacy in terms of complying to healthy lifestyle behaviors based on the median value of 19 units on the self-efficacy scale; Abbreviations—SD: Standard Deviation.

**Table 3:** OR and 95% CI evaluating the association between patients’ level of self-efficacy in terms of complying to healthy lifestyle behaviors (independent variable), both in total and separately in each psychosocial circumstance, with their level of adherence to the Mediterranean diet (high vs. low).

	OR	95%CI	*p*-Value
Level of self-efficacy in terms of complying to healthy lifestyle behaviors [high (Ref: low)]	1.72	1.45–2.04	<0.001
Level of self-efficacy for complying to healthy lifestyle behaviors (very confident vs. at most enough confident) when:			
You are out of home (invited on a social event, etc.)	1.61	1.33–1.92	<0.001
Your life is burdened with many obligations, and you don’t have time for yourself to exercise	1.45	1.20–1.72	<0.001
Others drink with no control	1.35	1.12–1.61	0.001
You have stress and negative feelings	1.27	1.05–1.52	0.011
Others eat food in the house that is not part of your diet	1.25	1.04–1.49	0.019
Your partner/friends smoke	1.11	0.93–1.33	0.276

Notes: Results are based on the multivariable logistic regression analysis, adjusting for patients’ demographic factors (age, sex), socioeconomic factors (educational level), and clinical characteristics (number of chronic conditions); level of adherence to the Mediterranean diet was estimated via the MedDietScore scale, and patients were categorized as low or high adherers based on the median value of 31 units on the scale; patients were categorized as having low or high self-efficacy in terms of complying to healthy lifestyle behaviors based on the median value of 19 units on the self-efficacy scale. Abbreviations—OR: Odds Ratio, CI = Confidence Interval, Ref: reference category.

**Table 4:** OR and 95% CI evaluating the association between patients’ level of self-efficacy in terms of complying to healthy lifestyle behaviors (independent variable), both in total and separately in each psychosocial circumstance, with their likelihood of being physically active (yes vs. no).

	OR	95%CI	*p*-Value
Level of self-efficacy in terms of complying to healthy lifestyle behaviors [high (Ref: low)]	1.30	1.09–1.56	0.004
Level of confidence for complying to healthy lifestyle behaviors (very confident vs. at most enough confident) when:			
You have stress and negative feelings	1.25	1.04–1.50	0.018
Your life is burdened with many obligations, and you don’t have time for yourself to exercise	1.19	1.01–1.43	0.046
You are out of home (invited on a social event, etc.)	1.16	0.96–1.40	0.124
Your partner/friends smoke	1.11	0.92–1.33	0.290
Others drink with no control	1.06	0.88–1.28	0.509
Others eat food in the house that is not part of your diet	1.01	0.84–1.22	0.885

Notes: Results are based on the multivariable logistic regression analysis, adjusting for patients’ demographic factors (age, sex), socioeconomic factors (educational level), and clinical characteristics (number of chronic conditions); patients were categorized as having low or high self-efficacy in terms of complying to healthy lifestyle behaviors based on the median value of 19 units on the self- efficacy scale. Abbreviations—OR: Odds Ratio, CI = Confidence Interval, Ref: reference category.

**Table 5:** OR and 95% CI evaluating the association between patients’ level of self-efficacy in terms of complying to healthy lifestyle behaviors (independent variable), both in total and separately in each psychosocial circumstance, with their likelihood of being non-smokers (yes vs. no).

	OR	95%CI	*p*-Value
Level of self-efficacy in terms of complying to healthy lifestyle behaviors [high (Ref: low)]	1.98	1.64–2.40	<0.001
Level of confidence in terms of complying to healthy lifestyle behaviors (very confident vs. at most enough confident) when:			
Your partner/friends smoke	5.88	5.00–7.69	<0.001
Your life is burdened with many obligations, and you don’t have time for yourself to exercise	1.61	1.32–1.96	<0.001
Others drink with no control	1.47	1.22–1.79	<0.001
You have stress and negative feelings	1.45	1.20–1.75	<0.001
You are out of home (invited on a social event, etc.)	1.32	1.08–1.61	0.007
Others eat food in the house that is not part of your diet	1.04	0.85–1.27	0.698

Notes: Results are based on the multivariable logistic regression analysis, adjusting for patients’ demographic factors (age, sex), socioeconomic factors (educational level), and clinical characteristics (number of chronic conditions); patients were categorized as having low or high self-efficacy in terms of complying to healthy lifestyle behaviors based on the median value of 19 units on the self- efficacy scale. Abbreviations—OR: Odds Ratio, CI = Confidence Interval, Ref: reference category.

## Data Availability

Data are available upon request. The data are not publicly available due to privacy or ethical restrictions.
